# Development of a gender-specific European job exposure matrix (EuroJEM) for physical workload and its validation against musculoskeletal pain

**DOI:** 10.5271/sjweh.4203

**Published:** 2025-03-01

**Authors:** Svetlana Solovieva, Alexis Descatha, Ingrid Sivesind Mehlum, Eira Viikari-Juntura, Karina Undem, Karin Berglund, Fabien Gilbert, Francesca Wuytack, Angelo d’Errico, Kathryn Badarin, Bradley Evanoff, Katarina Kjellberg

**Affiliations:** 1 Finnish Institute of Occupational Health, Helsinki, Finland.; 2 Univ Angers, CHU Angers, Univ Rennes, Inserm, EHESP, Irset (Institut de recherche en santé, environnement et travail) - UMR_S 1085, SFR ICAT, PCC- Prevention Federation, Angers, France.; 3 Epidemiology and Prevention, Donald and Barbara Zucker School of Medicine, Hofstra Univ Northwell Health, New York, USA.; 4 National Institute of Occupational Health (STAMI), Oslo, Norway.; 5 Department of Occupational and Environmental Medicine, Bispebjerg and Frederiksberg Hospitals, Copenhagen, Denmark.; 6 Department of Public Health, University of Copenhagen, Copenhagen, Denmark.; 7 Institute of Environmental Medicine, Karolinska Institutet, Stockholm, Sweden.; 8 The Centre for Occupational and Environmental Medicine, Region Stockholm, Stockholm, Sweden.; 9 Paris Cité University, “Population-based Cohorts Unit”, INSERM, Paris Saclay University, UVSQ, UMS 011, Paris, France.; 10 Department of Epidemiology, Local Health Unit ASL TO 3, Collegno, Turin, Italy.; 11 Division of General Medical Sciences, School of Medicine, Washington University in St. Louis, MO, USA.

**Keywords:** epidemiological study, harmonization, predictive validity

## Abstract

**Objectives:**

Develop a gender-specific European job exposure matrix (EuroJEM) for occupational physical workload and study its predictive validity for musculoskeletal pain in four European cohorts.

**Methods:**

National, gender-specific JEM from Finland, France, Norway and Sweden, based on self-reported exposure information, were evaluated for similarities in exposures, exposure definitions, and occupational coding. The EuroJEM harmonized five exposures: heavy lifting, faster breathing due to heavy workload, kneeling/squatting, forward bent posture, and working with hands above shoulder level. Our expert panel addressed disagreements and missing information to reach consensus on exposure levels across occupations. To assess predictive validity of the EuroJEM, we examined associations between the harmonized exposure measures and self-reported musculoskeletal pain across the four cohorts.

**Results:**

The EuroJEM provides semi-quantitative exposure estimates for 374 ISCO-88 (COM) occupational codes. Five categories of exposure were defined by the proportion of workers exposed within each occupation. Comparable and statistically significant associations were found between EuroJEM exposures and low back, shoulder, and knee pain across all cohorts and genders, except for knee pain among women in the Finnish cohort. For instance, in both genders heavy lifting, faster breathing due to heavy workload, and forward bent posture were statistically significantly associated with low-back pain in all four cohorts, with OR ranging from 1.25–2.18 (men) and 1.23–2.04 (women).

**Conclusions:**

Despite differences in study populations and outcome definitions, good predictive validity was observed in each national cohort, suggesting that EuroJEM can be an effective tool for exposure assessment in large-scale European epidemiological studies.

Job exposure matrices (JEM) can provide useful job exposure data in the absence of individual data. They provide exposure estimates based on job title and can be easily applied to cohorts and registries that include information on job titles or occupational codes. Additional advantages of JEM are their low cost and ability to reduce information bias, compared to self-reported exposures. This makes JEM very useful for research and surveillance (1, 2).

Over the last 40 years, several JEM have been developed for a range of different exposures, including physical workload. Generalizability and cross-national use of JEM have been explored by evaluating applicability of JEM developed in one country for use in another country (3–8).

A few efforts have been made to develop international JEM (9, 10). In general, combining JEM from different countries is a challenging task due to variation in occupational coding systems across the countries, in characterizations/definitions of exposures, and different metrics used in national JEM (11). Harmonization of existing JEM through features that are consistent and comparable can provide a standardized exposure measure across regions and time periods (12). Harmonized JEM may be useful for pooled analyses of cohorts from different countries, thereby increasing statistical power, and may contribute to increased generalizability of results across countries. The Exposome Project for Health and Occupational Research (EPHOR) seeks to construct a European JEM (EuroJEM) through harmonization of existing national JEM including a broad range of exposures, including physical workload, that can be used in pooled European cohorts (13)(www.ephor-project.eu).

JEM for physical workload have been developed in several European countries (14), and there is a large variation across the existing JEM in exposures and their definitions. Most existing JEM have been validated against self-reported exposures and have shown good predictive validity for different musculoskeletal outcomes (15–22).

We aimed to (i) develop a gender-specific EuroJEM for physical workload to be used for epidemiological studies in large European cohorts and (ii) examine its predictive validity for site-specific musculoskeletal pain in four European cohorts.

## Methods

JEM existing in Europe (see supplementary material, www.sjweh.fi/article/4203, table S1) were reviewed for similarities in included exposures, exposure definitions and assessments, and comparability of occupational coding systems. We selected the Finnish JEM for physical risk factors, the Norwegian mechanical JEM, the Swedish JEM (SWEJEM) for physical workload, and the French JEM CONSTANCES for harmonization. These JEM were gender-specific and provided information on the prevalence of exposures within the occupation.

*The Finnish JEM for physical risk factors* was developed utilizing self-reported exposure information from the large and nationally representative Health 2000 Survey, conducted in 2000–2001 (15). The matrix includes five exposures and covers 348 different occupational groups coded with the Finnish Standard Classification of Occupations 2001 coding system. The matrix showed a relatively high specificity without compromising sensitivity when compared with self-reported measures as well as a good predictive validity for low back pain (15).

*The Norwegian mechanical JEM* has been developed utilizing self-reported exposure information from the Norwegian nationwide Survey of Living Conditions on work environment, conducted in 2006 and 2009 (20). The matrix includes eight exposures and covers 268 different occupational groups coded with the Norwegian Standard Classification of Occupations (STYRK-98) coding system. The matrix showed overall fair-to-moderate agreement with self-reported exposures and a good predictive validity for low back pain (23).

*The SWEJEM for physical workload* has been constructed based on self-reported exposure information from the repeated Swedish Work Environment Surveys (SWES), conducted in 1997–2013 (21). The matrix includes eight exposures and covers 355 different occupational groups coded with the Swedish Standard Classification of Occupations (SSYK 96) coding system. The matrix showed a good predictive validity for frequent musculoskeletal pain (21).

*The French JEM CONSTANCES* has been developed based on self-reported exposure information of asymptomatic workers from the French nationally based CONSTANCES cohort between 2012–19 (22). People with musculoskeletal pain may overestimate workplace physical exposures (23), therefore, only responses of asymptomatic workers were used to generate JEM estimates. The matrix includes 27 physical workload exposures and covers 280 (for women) and 352 (for men) occupational groups coded with the French Classification of Occupations (Profession et Catégories Socioprofessionnelles PCS 2003). JEM CONSTANCES showed fair-to-moderate agreement with self-reported measures for most exposures, and odds ratios for musculoskeletal pain symptoms were similar to those using self-reported exposures (24).

Exposures included in the national JEM are listed in supplementary table S1.

### Validation cohorts

We used individual-level data of 25–60 years old men and women from four national population cohorts: the Health 2000 Study (H2000, Finland), the CONSTANCES cohort (France), the Surveys of Living Condition on Working Environment 2006 and 2009 (LKU 2006 and 2009, Norway), and the Stockholm Public Health Cohort (baseline 2002) (SPHC, Sweden).

In the H2000 Study national representative samples of the Finnish population aged 18–29 years and ≥30 years were obtained using a two-stage stratified cluster sampling design (25, 26). Subjects who were working during the past 12 months and had no missing data on occupation were included in the validation sample (N=3948).

The CONSTANCES cohort was designed as a representative sample of the salaried French or early retired adult population aged 18–69 (27). Randomly selected eligible participants completed self-administered questionnaires and underwent a health examination at one of 21 regional Health Screening Centers in 2012–2019. Subjects who were working at the time of recruitment and had no missing job or physical exposure data were included in the validation sample (N=104 910).

Statistics Norway carries out the LKU survey about every three years on a random sample of individuals aged 18–66, residing in Norway (28). For the current study, individuals participating in 2006 and/or 2009 were included (29). Subjects currently working and with no missing data on occupation were included in the validation sample (N=9534).

The SPHC is a population-based cohort that has conducted baseline surveys in the Stockholm region every four years since 2002. Participants are randomly selected from the adult population of Stockholm County (30). In the current study, the respondents of the baseline questionnaire 2002, who were currently working, and had no missing occupational code were included into the validation sample (N=12 759).

### Musculoskeletal symptoms

In the H2000 study, data on pain in the neck, shoulder, low back and different joints, including knees, were collected with questionnaire. The participants of the CONSTANCES cohort indicated on a pain diagram the presence of frequently occurring/daily pain in six body areas: hand/wrist, neck, shoulder, elbow, low back and knee/leg in the past 12 months and past 7 days. In LKU 2006 and 2009, data on pain in the low back, neck/shoulder, elbow/forearm/hands and hips/legs/knees/feet during the past month were collected through questionnaire. In the SPHC, data on daily pain in the low back and shoulder or arms during the past six months were collected by questionnaire at baseline 2002.

To test the predictive validity of the EuroJEM we selected five pain outcomes: (i) any pain in the low back during the past 30 days; (ii) frequently occurring/daily low back pain; (iii) frequently occurring/daily shoulder pain; (iv) any pain in the knee during the past 30 days; and (v) severe knee pain during the past 7 days. The selection was based on previous knowledge regarding the associations of these outcomes with the exposures included in EuroJEM. Another criterion was that the outcome was measured comparably in at least two cohorts (supplementary table S2).

### Development of the EuroJEM

The selected national JEM were examined in depth for similarities of included exposures and their definitions. We identified five relatively similarly defined exposures that were included in ≥2 of the national JEM: daily lifting >20 kg several times per day (heavy lifting), faster breathing due to physical workload, at least ¼ of the time (faster breathing), working in kneeling/squatting, at least ¼ of the time (kneeling or squatting), working in a forward bent posture, at least ¼ of the time (forward bent posture) and working with hands above shoulder level, at least ¼ of the time (hands above shoulder level) (supplementary table S3).

We harmonized the occupational codes into the same coding system [ISCO-88 (COM) - the European version of ISCO-88]. For this, the occupational codes of the three national JEM from the Nordic countries were transcoded to ISCO- 88 (COM) using recently developed Nordic crosswalks (31). The French PCS 2003 occupational codes were first transcoded to ISCO-88, using an earlier developed crosswalk (32) and then from ISCO-88 to ISCO-88 (COM).

**Figure 1:**
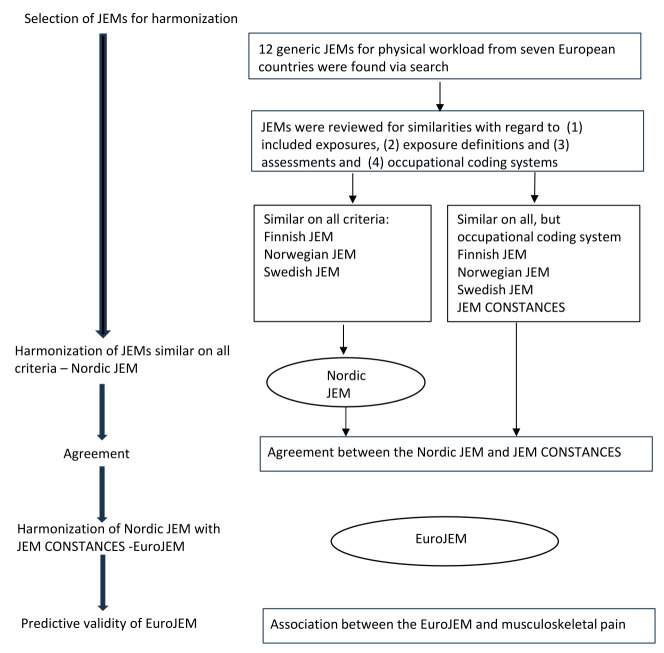
Flow chart for the development of EuroJEM.

The process of the EuroJEM development is summarized in figure 1. Due to unavailability of the gender-specific JEM CONSTANCES at the time when we started development of the EuroJEM and large differences between the French occupational coding system and ISCO-88, we started by harmonizing the Finnish, Norwegian and Swedish JEM (later called Nordic JEM) for four exposures. Then, we tested the agreement between the Nordic JEM and the gender-specific JEM CONSTANCES and harmonized the Nordic JEM and JEM CONSTANCES. By the time we started harmonization of the fifth exposure (hands above shoulder level), the gender-specific JEM CONSTANCES was available. Thus, we harmonized this exposure across all four national JEM at once.

Each selected exposure was harmonized within each occupation as follows. First, we categorized the proportion of exposed workers into five categories: 0=0–5%; 1=6–24%; 2=25–49%; 3=50–74%; 4=75–100%. Then, we compared and harmonized the exposure category for each occupation and gender in the national JEM. If the exposure category for an occupation was the same in all JEM, this exposure category was assigned to the occupation in the harmonized JEM. Disagreements between the national JEM were resolved by a consensus procedure of an expert panel. The panel consisted of two researchers from each country with expertise in occupational medicine and work environment. To resolve a disagreement, the panel discussed the tasks and activities within each occupation in question using descriptions of occupations and tasks in the national occupational classification manuals and took into consideration differences in exposure definitions between the JEM, possible estimation bias due to the small size of some occupations, and possible errors in exposure estimates due to the translation of the national occupational codes into the ISCO-88 (COM) code. In addition, the panelists also discussed possible regional and gender differences in exposure categories. The harmonization strategy is described in more detail in the supplementary material 3.

Concordance and agreement between the Finnish, Norwegian, Swedish and Nordic JEM is presented in supplementary material 4. Kappa values ranged from 0.39–0.72 depending on the national JEM and exposure. Kappa values were lower for forward bent posture than for the other exposures.

The EuroJEM provides five exposure categories based on the proportion of workers exposed. For testing the agreement between the Nordic JEM and JEM CONSTANCES, and the predictive validity of the EuroJEM, occupations were defined as non-exposed (0–24% workers exposed) and exposed (25–100% workers exposed).

### Statistical analyses

To assess agreement between the Nordic JEM and the JEM CONSTANCES, the exposure values from both JEM were assigned to the participants of the CONSTANCES cohort. We used the following indicators: (i) Cohen’s kappa coefficient to measure agreement and (ii) area under the receiver operating curve (AUC) to compare the ability of the JEM to classify exposed and non-exposed individuals. We interpreted Cohen’s kappa coefficient as: poor (<0.20), fair (0.21–0.40), moderate (0.41–0.60), good (0.61–0.80) and excellent (0.81–1) agreement. The AUC values were: failed (0.50–0.59), poor (0.60–0.69), fair (0.70–0.79), good (0.80–0.89) and excellent (0.90–1).

To test the predictive validity of the EuroJEM, we examined the associations of the EuroJEM exposure measures assigned to the participants in four different national cohorts with relevant site-specific musculoskeletal symptoms using logistic regression. The OR and their 95% confidence intervals (CI) were adjusted for age.

All analyses were performed within each population sample separately for men and women.

## Results

The EuroJEM included five physical workload exposures for 374 ISCO-88 (COM) codes. There were few occupations with ≥75% of workers being exposed (supplementary table S5). For the majority of occupational codes (N=242, 65%), men and women had similar exposure categories for all five exposures. Gender differences were most common for heavy lifting (58 codes) and least common for kneeling/squatting (9 codes). The number of occupational codes with gender differences in categories of exposures to faster breathing, forward bent posture, and hands above shoulder level were 32, 50 and 28, respectively. In 38 and 32 out of the 374 occupations, ≥25% of men were exposed to either all five or four out of five exposures, respectively. The corresponding numbers for women were 29 (five exposures) and 34 (four exposures).

### Agreement between the Nordic JEM and JEM CONSTANCES

Agreement between the Nordic JEM and the JEM CONSTANCES, assessed by kappa, ranged from fair to good for the four exposures examined (table 1). The agreement was better among women than men for all exposures except faster breathing. There was a good agreement for forward bent posture in both genders, for heavy lifting and kneeling/squatting among women, and faster breathing among men. Fair agreement was found for heavy lifting in men (kappa value 0.32) and for faster breathing in women (kappa value 0.39). For these two exposures there was a noticeable difference in prevalence of exposed workers between the two JEM. Among men, the prevalence of exposure to heavy lifting, estimated based on the Nordic JEM, was higher than that estimated based on JEM CONSTANCES (20.9% and 5.0%, respectively). Among women, the prevalence of exposure to faster breathing, estimated based on the Nordic JEM, was lower than that estimated based on JEM CONSTANCES (10.3% and 21.5%, respectively). The AUC was good to excellent for all exposures, except for faster breathing in women (0.62) and kneeling or squatting in men (0.76).

**Table 1:** Agreement measures between Nordic JEM and JEM CONSTANCES by gender.

Exposure	Men					Women			
	Kappa^a^	AUC	Proportion of exposed			Kappa	AUC	Proportion of exposed	
			Nordic JEM	JEM CONSTANCES				Nordic JEM	JEM CONSTANCES
Faster breathing	0.74	0.87	27.8	27.3		0.39	0.66	10.3	21.5
Heavy lifting	0.32	0.91	20.9	5.0		0.74	0.98	5.2	3.2
Kneeling or squatting	0.55	0.76	20.0	26.6		0.67	0.81	23.8	34.1
Forward bent posture	0.64	0.81	25.8	28.9		0.66	0.82	29.2	36.6

^a^ Cohen’s Kappa coefficient as follows: poor (<0.20), fair (0.21–0.40), moderate (0.41–0.60), good (0.61–0.80) and excellent (0.81–1) agreement. ^b^ AUC values were considered as follows: failed (0.50–0.59), poor (0.60–0.69), fair (0.70–0.79), good (0.80–0.89) and excellent (0.90–1).

### Description of validation cohorts

Table 2 shows descriptive characteristics of four national cohorts used for validation of the EuroJEM. The four cohorts differed by size of the study population, with the French cohort being the largest and the Finnish cohort the smallest. Both men and women in the Finnish cohort were younger than in the other cohorts. Manual workers were overrepresented in the Finnish cohort, as compared to the French, Norwegian and Swedish cohorts.

**Table 2:** Descriptive characteristics of the study populations and prevalence of exposures.

		Men												Women										
		Finnish (N=1927)			French (N=45 643)			Norwegian (N=4857)			Swedish (N=5774)			Finnish (N=2021)			French (N=59 267)			Norwegian (N=4677)			Swedish (N=6985)	
		Mean	%		Mean	%		Mean	%		Mean	%		Mean	%		Mean	%		Mean	%		Mean	%
Age (years)		42.1			43.0			43.5			44			42.6			43.0			43.2			44.0	
Major occupational groups																								
	Legislators, senior officials, and managers		16.7			11.8			14.6			11.3			7.1			6.9			6.8			5.3
	Professionals		16.8			31.1			18.6			30.6			19.0			30.1			22.8			31.0
	Technicians and associate professionals		13.8			22.0			22.5			23.9			18.9			30.3			25.9			26.9
	Clerks		1.9			5.0			4.2			4.6			13.9			13.8			8.6			14.0
	Service and sales workers		4.7			5.4			8.1			5.8			22.4			10.7			27.5			17.7
	Skilled agricultural and fishery workers		7.1			0.6			1.0			0.8			3.3			0.2			0.4			0.3
	Craft and related trades workers		22.5			11.4			15.8			13.7			2.7			1.1			1.1			1.0
	Plant and machine operators and assemblers		11.8			8.3			11.0			7.3			3.4			1.4			1.9			1.7
	Elementary occupations		4.9			4.2			4.2			2.0			9.5			5.6			5.2			2.2
Heavy lifting																								
	Prevalence >25%		34.3			16.1			22.2			16.7			16.5			5.3			16.6			7.7
Faster breathing																								
	Prevalence >25%		43.4			28.2			32.5			23.4			24.9			14.1			18.2			11.6
Forward bent posture																								
	Prevalence >25%		41.5			26.2			32.0			25.9			38.2			34.3			38.1			28.4
Hands above shoulder level																								
	Prevalence >25%		33.9			17.3			22.3			18.1			16.2			9.0			7.3			5.2
Kneeling/squatting																								
	Prevalence >25%		32.8			18.7			22.6			17.0			27.1			23.2			30.7			22.1

Among men, the prevalence of all five exposures was highest in the Finnish cohort (table 2). Differences in the prevalence of exposures between French and Swedish cohorts were small. Among women the prevalence of most of the exposures was similar between the Norwegian and Finnish cohorts, and between the Swedish and French cohorts.

In all four cohorts, the prevalence of musculoskeletal symptoms was higher among women than men (table 3). In both genders, the prevalence of low-back pain during the past 30 days was higher in the Norwegian cohort than Finnish cohort. Prevalence of frequently occurring or daily pain in the low back and shoulder was higher in the French than Swedish cohort. The prevalence of knee pain during the past 30 days in the Finnish cohort and severe knee pain in the French cohort was similar.

**Table 3:** Prevalence of pain outcomes by gender and by national cohort

			Finnish		Norwegian		French		Swedish
			N (%)		N (%)		N (%)		N (%)
Men									
	Low-back pain								
		Any pain in past 30 days	528 (27.4)		1636 (33.7)				
		Frequently occurring/daily					4032 (13.1)		275 (4.8)
	Shoulder pain								
		Frequently occurring/daily					2359 (10.9)		337 (5.8)
	Knee pain								
		Any pain in past 30 days	281 (14.6)						
		Severe pain in past 7 days					2941 (13.8)		
Women									
	Low-back pain								
		Any pain in past 30 days	612 (30.3)		1901 (40.7)				
		Frequently occurring/daily					6293 (15.3)		453 (6.5)
	Shoulder pain								
		Frequently occurring/daily					4302 (13.9)		665 (9.5)
	Knee pain								
		Any pain in past 30 days	339 (16.8)						
		Severe pain in past 7 days					4480 (14.4)		

### Predictive validity of the EuroJEM

In both genders, heavy lifting, faster breathing and forward bent posture were statistically significantly associated with low-back pain in all four cohorts (table 4). The OR were in the range of 1.25–2.18 among men and 1.23–2.04 among women. In both genders, heavy lifting, faster breathing and hands above shoulder level were statistically significantly associated with frequently occurring/daily shoulder pain in the French and Swedish cohorts, with OR being in the range of 1.43–1.85 among men and 1.61–2.32 among women. Heavy lifting and kneeling/squatting were statistically significantly associated with severe knee pain among women and men in the French cohort (OR in the range of 1.39–1.93, being lower among women than men) and pain in the knee during the past 30 days only among men in the Finnish cohort (OR being 1.58 and 1.75, respectively).

**Table 4:** Associations between JEM-based exposures and musculoskeletal pain by gender and national cohort. [CI=confidence interval.]

			Finnish		Norwegian			French			Swedish
			OR (95% CI)		OR (95% CI)			OR (95% CI)			OR (95% CI)
**Men**											
Low-back pain											
	Any pain in past 30 days										
		Heavy lifting	1.53 (1.24-1.88)		1.48 (1.28-1.70)						
		Faster breathing	1.40 (1.15-1.71)		1.35 (1.19-1.53)						
		Forward bent posture	1.25 (1.02-1.53)		1.34 (1.18-1.52)						
	Frequently occurring/daily										
		Heavy lifting						1.94 (1.79-2.09)			1.78 (1.35- 2.35)
		Faster breathing						1.90 (1.77-2.03)			2.18 (1.69-2.80)
		Forward bent posture						1.71 (1.59-1.83)			1.88 (1.46- .2.41)
Shoulder pain											
	Frequently occurring/daily										
		Heavy lifting						1.55 (1.37-1.74)			1.43 (1.09-1.87)
		Faster breathing						1.82 (1.68-1.97)			1.76 (1.42-2.20)
		Hands above shoulder level						1.78 (1.62-1.95)			1.85 (1.36-2.52)
Knee pain											
	Any pain in past 30 days										
		Heavy lifting	1.58 (1.22-2.04)								
		Kneeling/squatting	1.75 (1.35-2.26)								
	Severe pain in past 7 days										
		Heavy lifting						1.93 (1.77-2.00)			
		Kneeling/squatting						1.82 (1.67-1.98)			
**Women**											
Low-back pain											
	Any pain in past 30 days										
		Heavy lifting	1.36 (1.06-1.74)		1.57 (1.35-1.84)						
		Faster breathing	1.31 (1.06-1.63)		1.54 (1.32-1.79)						
		Forward bent posture	1.23 (1.01-1.50)		1.31 (1.16-1.48)						
	Frequently occurring/daily										
		Heavy lifting						1.58 (1.43-1.75)			1.52 (1.11-2.08)
		Faster breathing						2.04 (1.90-2.17)			1.81 (1.41- 2.33)
		Forward bent posture						1.31 (1.24-1.38)			1.24 (1.01- 1.53)
Shoulder pain											
	Frequently occurring/daily										
		Heavy lifting						1.88 (1.70-2.07)			2.05 (1.60-2.32)
		Faster breathing						1.78 (1.63-1.94)			2.32 (1.84-2.92)
		Hands above shoulder level						1.61 (1.46-1.78)			1.96 (1.53-2.51)
Knee pain											
	Any pain in past 30 days										
		Heavy lifting	1.19 (0.88-1.61)								
		Kneeling/squatting	1.14 (0.88-1.48)								
	Severe pain in past 7 days										
		Heavy lifting						1.63 (1.44-1.83)			
		Kneeling/squatting						1.39 (1.29-1.49)			

## Discussion

We developed a gender-specific European JEM for physical workload factors by harmonizing existing national JEM from Finland, France, Norway, and Sweden and examined predictive validity for relevant musculoskeletal pain, utilizing data from four national cohorts. The matrix was designed for use in large-scale epidemiological studies as a tool for assessment of physical workload. The EuroJEM included the following five physical workload factors: heavy lifting, faster breathing, forward bent posture, hands above shoulder level, and kneeling/squatting. The occupation axis of the matrix was based on the ISCO-88 (COM) codes. The strongest agreements were observed for heavy lifting among women and faster breathing among men. Conversely, the weakest agreements were found for the same exposures but for the opposite genders: heavy lifting among men and faster breathing among women. All five exposures included in the EuroJEM showed good predictive validity for relevant site-specific musculoskeletal pain (low back, shoulder, and/or knee pain) among both men and women.

JEM for physical workload are often constructed utilizing self-reported information from nationally representative surveys. Currently, there is a growing interest in applying JEM for exposure assessment in cross-national studies. Up until now, few studies have compared general population JEM across different countries; existing data suggest that some exposure estimates based on industry and job title are comparable across countries, while others are not: Lavoué and colleagues observed moderate to very good agreement of exposure estimates for several chemicals when comparing Finnish and Canadian JEM (33). Findings from another study suggested that better estimates for exposure-outcome associations could be achieved by combining exposures from different JEM (34). However, the JEM developed in different countries are not identical, and while they are usually valid in the settings where they have been developed, their performance might differ across different countries (11). In addition to differences in exposure types and definitions, cross-national JEM differ in occupational classification codes used to assign the exposure estimates to occupations, which may introduce misclassification of JEM estimates due to imperfect matches of occupations.

The four national JEM included in this harmonization were selected out of several physical workload JEM existing in Europe, based on their similarity in the metrics and comparability in exposure definitions and assessment, as well as comparability of occupational coding systems. However, the national occupational classification systems of the selected JEM still differed, with the largest difference being between the French PCS 2003 and the ISCO-based classifications used by the three Nordic countries.

Even though the exposures were comparable across the selected national JEM, they were not identical. JEM CONSTANCES did not include the exposure faster breathing due to heavy workload, so this exposure was estimated by Borg’s rating of perceived exertion scale. In the Swedish and Norwegian JEM, this exposure was assessed by the duration of performing work leading to faster breathing using six- and five-point ordinal scales, respectively. This exposure was not included in the Finnish JEM. Heavy lifting was also defined somewhat differently in each national JEM. The JEM used different load weights (10–25 kg) as well as different frequency of lifting to define exposure to heavy lifting. Furthermore, among men, the prevalence of heavy lifting based on JEM CONSTANCES was much lower than the prevalence based on the Nordic JEM, while among women it was similar. Relatively small proportions of CONSTANCES participants (10.5%) were employed in manual occupations (eg, agriculture, construction) (35), suggesting that the prevalence of exposure in the JEM CONSTANCES among men was underestimated as compared to general French working population. Nevertheless, our results suggest that exposures assessed by these two different JEM were related and could be incorporated into one JEM.

We examined the predictive validity of the EuroJEM for musculoskeletal pain by estimating associations of site-specific pain with exposures that have been linked with these outcomes in previously published studies. We utilized data from four national cohorts and picked relatively similar self-reported outcomes (both prevalent and more severe pain) being included in at least two cohorts to compare the observed associations between the cohorts. Observed associations of the EuroJEM-based exposures with low back, shoulder and knee pain were in line with previously published findings. For example, similar associations were found in meta-analyses between low-back pain and lifting/carrying loads (OR 1.7, 95% CI 1.4–2.2), and between low-back pain and non-neutral postures (OR 1.5, 95% CI 1.2–1.9) (36). A meta-analysis on shoulder disorders found an association between shoulder disorders and hand-arm elevation (OR 1.9, 95% CI 1.47–2.47) (37), and another meta-analysis found associations of knee disorders with kneeling 1.29 (95% CI 1.05–1.57) and lifting 1.39 (95% CI 1.22–1.59) (38). The only statistically non-significant associations were found for heavy lifting and kneeling/squatting with knee pain among women in the Finnish cohort. One possible explanation for the weak associations could be that a relatively small sample size resulted in insufficient statistical power. Nevertheless, the associations were reproducible and comparable across cohorts, regardless of the differences in the composition of study populations and the definition of the outcomes. Thus, our results provide evidence for good predictive validity of the EuroJEM.

Some limitations should be mentioned. First, all JEM have general limitations by design, which will be retained in the harmonized JEM. By assigning the same exposure to all workers in a job, JEM reflect an “average” level of exposure, which cannot account for exposure heterogeneity among individual workers in the same job (12, 39). Additionally, the expertise and opinions of the experts during the harmonization process may influence the exposure levels assigned in the JEM and thus can result in exposure misclassification (12). Nevertheless, JEM have been widely used for exposure assessment in large populations, and comparable results across different cohorts found in the current study are promising. Second, we used data from only four countries. Each of these cohorts has limitations. For example, the CONSTANCES and Stockholm Public Health Cohorts are not fully representative of their national workforces, and three of the four cohorts are from Nordic countries. However, previous studies based on these cohorts have confirmed the validity of analyses of musculoskeletal risk factors and outcomes (15, 20, 21, 25). However, substantial similarity of exposures seen between Nordic countries and France suggest that the EuroJEM can be used in other countries. Similarly, the American O*NET JEM has shown predictive validity in the UK, France, and Italy (6, 40, 41). Third, even though data material for the harmonized variables in the included national JEM was collected between 1997 and 2019, only one average measure for the time period covered in each JEM and for each exposure was available. As there is little evidence of recent large-scale changes in physical workload exposures, we believe that this JEM can reasonably be used to estimate exposures for the past few decades and the near future. Additional studies should compare changes in reported exposures over time to allow better estimates of exposure variation across time periods. Last, semi-quantitative metrics for exposure categories were chosen in order to obtain better agreement across different countries and different coding systems.

Despite these limitations, it is important to highlight the strengths of the developed EuroJEM for physical workload. The creation of a cross-national and gender-specific JEM, harmonized by experts and validated in four different cohorts with similar results, is unique. This EuroJEM holds great potential for a wide range of research and public health applications. By enabling the use of large cohorts and existing administrative data, JEM can facilitate the study of work-related health outcomes, even when exposure data is limited to job title, occupational code, or job history. This can be especially valuable for public health researchers outside the field of occupational health, providing a simple and inexpensive method to incorporate occupational exposures into their analyses. Additionally, physical workload factors included in the EuroJEM may be predictive of conditions beyond musculoskeletal disorders. For example, work requiring physical exertion (faster breathing) may be relevant for cardiovascular outcomes (42). Further research is needed to extend the EuroJEM to other relevant exposures (eg, repetitive and forceful hand /arm movements, standing and walking) and to confirm applications in different contexts.

In conclusion, our results showed that the Nordic JEM and JEM CONSTANCES could be incorporated into a unified EuroJEM. The EuroJEM showed a good predictive validity in different national cohorts, despite differences in the study populations and outcome definitions. Future research in diverse settings and with different outcomes will be valuable, as will the application of the EuroJEM to cohorts from other European countries and beyond.

## Supplementary material

Supplementary material
