# Supplementary material for: Development of a gender-specific European job exposure matrix (EuroJEM) for physical workload and its validation against musculoskeletal pain
Source: Scand J Work Environ Health. 2025 Feb 28;51(2):119–29. doi: 10.5271/sjweh.4203 (PMC11895771; doi:10.5271/sjweh.4203)
Supplement: Supplementary material [file SJWEH-51-119-S001.pdf]

Development of a gender-specific European job exposure matrix (EuroJEM) for physical workload and its validation against musculoskeletal pain[1]

by Svetlana Solovieva, PhD, Alexis Descatha, MD, PhD, Ingrid Sivesind Mehlum, MD, PhD, Eira Viikari-Juntura, MD, PhD, Karina Undem, MPhil, Karin Berglund, Mpt, Fabien Gilbert, MSc, Francesca Wuytack, PhD, Angelo d'Errico, MD, Kathryn Badarin, PhD, Bradley Evanoff, MD, MPH, Katarina Kjellberg, PhD[2]

1. SUPPLEMENTARY MATERIAL
2. Correspondence to: Katarina Kjellberg, Institute of Environmental Medicine, Karolinska Institutet, Stockholm, Sweden. [E-mail: katarina.kjellberg@ki.se]

SUPPLEMENTARY MATERIAL 1

The existing JEMs in Europe were identified via search of published documents in PubMed and Google Scholar, complemented by JEMs registered in the JEM inventory of the OMEGA-NET COST Action project (https://occupationalexposuretools.net/). In total 12 generic JEMs for physical workload from seven countries were found (Supplementary Table S1). The majority of the JEMs used national occupational classification systems based on the International Standard Classification of Occupations 1988 (ISCO-88). In order to select JEMs and exposures for harmonization, all identified JEMs were reviewed for similarities in included exposures, exposure definitions and assessments, and comparability of occupational coding systems.

Table S1. Overview of existing generic JEMs for physical workload

JEM name, country	Exposures	Exposure metric	Occupational codes
Expert-based			
Lower body JEM, Denmark[1]	Lifting, kneeling/squatting, standing/walking and sitting	Mean score of expert's ratings	DISCO-88, comparable with ISCO-88
Shoulder JEM, Denmark[2]	Force exerted with the hand and arm, upper-arm elevation >90°, moderately and highly repetitive work.	Mean score of expert's ratings.	DISCO-88, comparable with ISCO-88
	A summary shoulder workload	Mean score	
MADE, France[3]	Physical effort, heavy lifting, very heavy lifting, static strength, dynamic strength, kneeling or squatting, cervical constraints, shoulder abducted >90°, elbow constraints, turn the hand (e.g. screwing), repetition, press base of hand, grip.	Mean score of expert's ratings.	French 4-digit Profession et Catégorie Sociale (PCS) job code
Based on self-reports			
Physical JEM, Denmark[4]	Physical work demands index, including the following exposures: sitting, walking or standing, working with the back twisted or bent, arms lifted above the shoulders, repetitive arm movements, squatting kneeling, pushing or pulling and carrying or lifting.	Mean score of Likert scale's categories	DISCO-08, comparable with ISCO-08

JEM for physical workload, Finland[5]	Heavy physical work, heavy lifting, kneeling/squatting, working with hands above shoulder level, working in forward bent posture.	Proportion of exposed	National occupational classification, comparable with ISCO-88
FINJEM, Finland[6]	Perceived physical workload, inconvenient and difficult work postures, manual handlings of burdens, repetitive work involvement, sedentary work, standing work.	Proportion of exposed	Own classification, based on ISCO-58
JEM CONSTANCES, France[7]	Physical intensity of work, carry loads (< 10 kg; 10-25 kg, > 25 kg), handle objects 1–4 kg, >4 kg, kneeling/squatting, working with arms above shoulder level, bending trunk, bending neck, reaching behind, working with arms abducted, bending elbow, rotating forearm, bending wrist, pressing base of hand, finger pinching, working with repetition, standing and changing tasks.	Mean, median and bias corrected mean min/day	French 4-digit Profession et Catégorie Sociale (PCS) job code
Gender-specific JEM CONSTANCES, France[8]	Similar as above	Proportion of exposed	French 4-digit Profession et Catégorie Sociale (PCS) job code
GPJEM, The Netherlands[9]	Force (in lifting, pushing, pulling or carrying or using force with work tools), work in uncomfortable positions and repetitive movements.<br>A summary physical demands score.	Exposure categories based on proportion of exposed.	NSCO-92, can be linked to ISCO-08
Mechanical JEM, Norway[10]	Fast breathing due to physical workload, heavy lifting, kneeling/squatting, working with hands above shoulder level, working in forward bent posture, work with neck flexion, awkward lifting and standing/walking.	Proportion of exposed	STYRK-98, comparable with ISCO-88
MatEmEsp, Spain[11]	Perceived physical workload, inconvenient and difficult work postures, manual handlings of burdens, repetitive work involvement, sedentary work, standing work.	Proportion of exposed	CNO-94, a national system based on ISCO-88
SWEJEM, Sweden[12]	Heavy lifting (≥15kg), physically strenuous work, fast breathing due to heavy workload, forward bent position, twisted position, working with hands above shoulder level, repetitive work and frequent bending and twisting.<br>Physical workload index	Proportion of exposed<br>Mean score	SSYK -96, comparable with ISCO-88

References

1. Rubak TS, Svendsen SW, Andersen JH, Haahr JP, Kryger A, et al. An expert-based job exposure matrix for large scale epidemiologic studies of primary hip and knee osteoarthritis: the Lower Body JEM. BMC Musculoskelet Disord. 2014; 15:204.
2. Svendsen SW, Dalbøge A, Andersen JH, Thomsen JF, Frost P. Risk of surgery for subacromial impingement syndrome in relation to neck-shoulder complaints and occupational biomechanical exposures: a longitudinal study. Scand J Work Environ Health .2013; 39:568-77.
3. Descatha A, Despréaux T, Petit A, Bodin J, Andersen JH, et al. Development of "MADE", a French Job exposure matrix for evaluation of biomechanical exposure (in French). Sante Publique. 2018; 30:333-7.
4. Madsen IEH, Gupta N, Budtz-Jørgensen E, Bonde JP, Framke E, et al. Physical work demands and psychosocial working conditions as predictors of musculoskeletal pain: a cohort study comparing self-reported and job exposure matrix measurements. Occup Environ Med. 2018; 75:752-8.
5. Solovieva S, Pehkonen I, Kausto J, Miranda H, Shiri R, et al. Development and validation of a job exposure matrix for physical risk factors in low back pain. PLoS One. 2012; 7:e48680.
6. Kauppinen T, Toikkanen J, Pukkala E. Am From cross-tabulations to multipurpose exposure information systems: a new job-exposure matrix. J Ind Med. 1998; 33:409-17.
7. Evanoff BA, Yung M, Buckner-Petty S, Andersen JH, Roquelaure Y, et al. The CONSTANCES job exposure matrix based on self-reported exposure to physical risk factors: development and evaluation. Occup Environ Med. 2019; 76:398-406.
8. Wuytack F, Evanoff BA, Dale AM, Gilbert F, Fadel M, Leclerc A, Descatha A. Development and evaluation of the gender-specific CONSTANCES job exposure matrix for physical risk factors in France. Scand J Work Environ Health. 2023; 49:549-557.
9. Hanvold TN, Sterud T, Kristensen P, Mehlum IS. Mechanical and psychosocial work exposures: the construction and evaluation of a gender-specific job exposure matrix (JEM). Scand J Work Environ Health 2019; 45:239-247.
10. Rijs KJ, van der Pas S, Geuskens GA, Cozijnsen R, Koppes LL, et al. Development and Validation of a Physical and Psychosocial Job-Exposure Matrix in Older and Retired Workers. Ann Occup Hyg. 2014; 58:152-70.
11. García AM, González-Galarzo MC, Kauppinen T, Delclos GL, Benavides FG. A job-exposure matrix for research and surveillance of occupational health and safety in Spanish workers: MatEmESp. Am J Ind Med. 2013; 56:1226-38.
12. Badarin K, Hemmingsson T, Hillert L, Kjellberg K. Physical workload and increased frequency of musculoskeletal pain: a cohort study of employed men and women with baseline occasional pain. Occup Environ Med. 2021; 78:558-66.

SUPPLEMENTARY MATERIAL 2.

Table S2. Assessment of musculoskeletal pain in four European cohorts

Body area		Outcomes used in the study	Health 2000 Study, Finland	LKU Survey, Norway	CONSTANCES cohort, France	SPHC, Sweden
Low back						
	Question		Have you during the past month (30 days) had a low back pain? Yes/no	During the past month, have you been bothered by pain in the lower back or the lower part of the back? 1=very much; 2=quite; 3= a little; 4=not at all		
	Definition	any pain during the past 30 days	low back pain during the past 30 days	any pain (1-3 categories) in the low back during the past month		
					Over the past 12 months, have you experienced any problems (aches, pains, discomfort, numbness) in the low back? Yes/no If yes, how long you suffered? < 24 hours; 1 to 7 days; 8 to 30 days; > 30 days; constantly	Have you had any pain in the lower back in the past 6 months? 0=No, never; 1= yes, a couple of days during the last year; 2=yes, a couple of days per month; 3=yes, a couple of days per week; 4=yes, every day
		frequently occurring/daily			constantly occurring problems in the low back during the past 12 months	daily in the lower back in the past 6 months
Shoulder						
	Question				Over the past 12 months, have you experienced any problems (aches, pains, discomfort, numbness) in the shoulder? Yes/no How long you suffered? < 24 hours; 1 to 7 days; 8 to 30 days; > 30 days; constantly	Have you had any pain in the shoulders or arms in the past 6 months? 0=No, never; 1= yes, a couple of days during the last year; 2=yes, a couple of days per month; 3=yes, a couple of days per week; 4=yes, every day
	Definition	frequently occurring/daily			constantly occurring problems in the low back during the past 12 months	daily in the lower back in the past 6 months
Knee						
	Question		Have you during the previous month (30 days) had pain, ache or motion soreness in one or both knee joints? Yes/no			
	Definition	any pain during the past 30 days	pain in the knee joints during previous month			
	Question				Over the past 7 days, have you experienced any problems (aches,	

		pains, discomfort, numbness) in the knee/leg? Yes/no<br>If yes, how intensive pain was?<br>0 (no pain) - 10
Definition	severe pain during the past 7 days	Knee pain of intensity above 5 points during the past 7 days

SUPPLEMENTARY MATERIAL 3. Definitions of exposures selected for harmonization and the harmonization procedure

Table S3. Definition of exposures selected for harmonization.

Exposure		National JEMs				EuroJEM
		Finnish	French	Norwegian	Swedish	
Heavy lifting						
	Definition	Lifting > 20 kg at least 10 times per day, every day. Response categories: yes/no	Lifting and carrying a load that weighs > 10-25 kg Lifting and carrying a load that weighs > 25 kg Response categories (in each question): Never or nearly never; <2 hours per day; 2-4 hours per day; > 4 hours per day	Lifting > 20 kg Response categories: > 20 times/ day; 5-19 times/day; 1-4 times/ day; not at all	Lifting > 15 kg several times per day Response categories: every day; couple of days/week; one day/week; a couple of days/month; not at all/rarely	Daily lifting >20 kg
	Exposed	at least 10 times per day, every day	At least > 4 hours per day	5-19 times per day, every day	Several times per day, every day	Several times per day, every day
	Metric	Proportion of exposed, cont.	Proportion of exposed, cont.	Proportion of exposed, cont.	Proportion of exposed, cont.	Proportion of exposed, 5 categories
Fast breathing due to heavy workload						
	Definition		Borg Rating of Perceived Exertion (RPE). Scale from 6 (no effort at all. sitting and doing nothing) to 20 (absolute maximal effort, highest possible exhaustion)	Faster breathing due to physical workload. Response categories: almost all the time; ¾ of the workday; ½ of the workday; ¼ of the workday; very little of the workday	Faster breathing due to physical workload. Response categories: almost all the time; about ¾ of the time; half of the time; about ¼ of the time; about 1/10 of the time; not at all	Faster breathing due to physical workload
	Exposed		Borg RPE Scale > 15 (at least strong effort needed)	¼ of the workday or more	¼ of the time or more	¼ of the time or more
	Metric		Proportion exposed	Proportion exposed	Proportion exposed	Proportion of exposed, 5 categories
Kneeling/squatting						
	Definition	Kneeling or squatting for at least one hour a day. Response categories: yes/no	Kneeling or squatting during a typical workday: Never or nearly never; <2	Daily kneeling/ squatting. Response categories:		Daily kneeling or squatting

			hours per day; 2-4 hours per day; > 4 hours per day	almost all the time; ¾ of the workday; ½ of the workday; ¼ of the workday; very little of the workday		
	Exposed	One hour a day or more	2-4 h or more/day	¼ of the workday or more		¼ of the time or more
	Metric	Proportion exposed	Proportion exposed	Proportion exposed	Proportion exposed	Proportion of exposed, 5 categories
Forward bent posture						
	Definition	Working in forward bent posture (while standing or kneeling) for at least one hour a day,<br>Response categories: yes/no	Do you lean forward or sideways regularly or for prolonged periods?<br>Response categories: Never or nearly never; <2 hours per day; 2-4 hours per day; > 4 hours per day	Working in forward bent posture (without supporting on hands or arms).<br>Response categories: almost all the time; ¾ of the workday; ½ of the workday; ¼ of the workday very little of the workday	Working in forward bent posture (while not using support from hands or arms).<br>Response categories: almost all the time; about ¾ of the time; half of the time; about ¼ of the time; about 1/10 of the time; not at all	Working in a forward bent position
	Exposed	One hour a day or more	2-4 hours per day or more	¼ of the workday or more	¼ of the time or more	¼ of the time or more
	Metric	Proportion exposed	Proportion exposed	Proportion exposed	Proportion exposed	Proportion of exposed, 5 categories
Hands above shoulder level						
	Definition	Working with hands above shoulder level for at least one hour a day.<br>Response categories: yes/no	Daily working with one or two arms above shoulders level regularly or for a prolonged period.<br>Response categories: never or nearly never; <2 hours per day; 2-4 hours per day; > 4 hours per day	Working with hands at or above shoulder level, daily.<br>Response categories: almost all the time; ¾ of the workday; ½ of the workday; ¼ of the workday; very little of the workday	Sometimes working with hands at or above shoulder level.<br>Response categories: almost all the time; about ¾ of the time; half of the time; about ¼ of the time; about 1/10 of the time; not at all	Working with hands above shoulder level
	Exposed	One hour a day or more	2-4 h or more/day	¼ of the workday or more	¼ of the time or more	¼ of the time or more
	Metric	Proportion exposed	Proportion exposed	Proportion exposed	Proportion exposed	Proportion of exposed, 5 categories

Harmonization procedure

1. For each selected exposure in the national JEMs we categorized the proportion of exposed into five categories: 0=0-5%; 1=6-24%; 2=25-49%; 3=50-74%; 4=75-100%.
2. The occupations were checked for agreement regarding exposure category between the JEMs.
   - When there **was full** agreement between JEMs for an occupation, this category value was assigned to the EuroJEM.
   - For occupations with **disagreement**s, the expert panel discussed until a consensus was reached, and then assigned exposure categories to the EuroJEM.

   The following considerations were used in the consensus discussions:

   a. Tasks and activities within the occupation were discussed and used to resolve disagreements. Correspondence between the descriptions of the occupation under ISCO-88(COM) code and for matched national codes were also looked at. Discussions were held about whether the exposure categories of the different JEMs were reasonable according to what was known by the panel experts regarding work tasks and activities.

   b. Characteristics of the national JEMs:

      i. The size of the occupational group, i.e. the number of respondents from the particular occupation in the survey on which the exposure estimate in the national JEM was calculated. The following categories for the size of occupations were used: >199, 50-199, 10-49 and < 10. JEMs where the estimate was based on smaller group sizes were considered less reliable. Estimates from JEMs with larger sizes had higher weights for the consensus agreements.

      ii. Attention was paid to, whether there was a genuine estimate (based on survey responses for the specific occupation and gender) or whether the estimate was based on merged occupations or genders. JEMs with genuine estimates were considered more reliable.

      iii. For the exposure “heavy lifting”, the size of loads is assessed differently in the JEMs of the Nordic countries (as described above). As heavy lifting is defined as lifting > 20 kg in both the Norwegian and Finnish JEMs, this size of load was used for the EuroJEM. However, in the Swedish JEM, heavy lifting is defined with a lower cut-off load: > 15 kg. We found that for several occupations, the proportion exposed to heavy lifting was higher in the Swedish than in the Norwegian or Finnish JEMs. In such cases it was assumed that the exposure value was high due to the lower load size in the Swedish JEM. However, for some occupations, the proportion of workers exposed to heavy lifting in the Swedish JEM was lower than in either the Norwegian or Finnish JEM. In such cases, if the size of occupation in the Swedish JEM was large, the estimates of the Swedish JEM had a higher weight in resolving the disagreement.

   c. If disagreements were considered to reflect true differences between the countries regarding the work content in an occupation, and all values to be trustworthy with regard to i - iii. above, a mean value of the exposure categories was assigned. If one national value was close to the border of the exposure category, and close to the other JEM values, this was also taken into consideration.

3. For occupations with missing information on exposure estimates from most of the national JEMs, the expert panel discussed until reaching a consensus and assigned exposure categories to these

occupations. The same considerations described above (bullet 3) were used, with the addition of the following solutions when relevant:

a. For occupations with agreement in exposure rates based on two JEMs and missing in the third JEM, the exposure category from the two JEMs was assigned.

b. When exposure estimates were available from at least one national JEM, the exposure category from a JEM with a relatively large occupation size was assigned.

c. If an exposure estimate was available for only one gender, it was considered to assign the same exposure category for both genders (a similar strategy was applied during the construction of the national JEMs).

SUPPLEMENTARY MATERIAL 4. Concordance and agreement between the Finnish, Norwegian, Swedish and the Nordic JEM.

Table S4.1. Concordance of exposure metrics between the Finnish, Norwegian, Swedish and the Nordic JEM. Number of ISCO-88 (COM) codes by exposure category among men and women.

	Men				Women			
Exposure/categories	Finnish JEM	Norwegian JEM	Swedish JEM	Nordic JEM	Finnish JEM	Norwegian JEM	Swedish JEM	Nordic JEM
Heavy lifting	N=351	N=284	N=330	N=374	N=347	N=264	N=325	N=374
0-5%	98 (27.9%)	113 (39.8%)	125 (37.9%)	138 (36.9%)	123 (35.4%)	125 (47.3%)	153 (47.1%)	170 (45.5%)
6-24%	143 (40.7%)	114 (40.1%)	87 (26.4%)	131 (35.0%)	158 (45.5%)	99 (37.5%)	108 (33.2%)	140 (37.4%)
25-49%	88 (25.1%)	46 (16.2%)	84 (25.5%)	91 (24.3%)	53 (13.3%)	31 (11.7%)	54 (16.6%)	62 (16.6%)
50-74%	14 (4.0%)	11 (3.9%)	27 (8.2%)	12 (3.2%)	11 (3.2%)	8 (3.0%)	10 (3.1%)	2 (0.5%)
75-100%	8 (2.3%)	0 (0.0%)	7 (2.1%)	2 (0.5%)	2 (0.6%)	1 (0.4%)	0 (0.0%)	0 (0,0%)
Fast breathing		N=280	N=314	N=374		N=270	N=312	N=374
0-5%		77 (27.5%)	92 (29.3%)	110 (29.4%		83 (30.7%)	126 (40.4%)	124 (33.2%
6-24%		130 (46.4%)	102 (32.5%)	135 (36.1%)		116 (43.0%)	109 (34.9%)	138 (36.9%)
25-49%		56 (20.0%)	86 (27.4%)	98 (28.2%)		54 (20.0%)	59 (18.9%)	89 (23.8%)
50-74%		17 (6.1%)	33 (10.5%)	31 (8.3%)		17 (6.3%)	15 (4.8%)	23 (6.1%)
75-100%		0 (0.0%)	1 (0.3%)	0 (0.0%)		0 (0.0%)	3 (1.0%)	0 (0.0%)
Forward bent posture	N=350	N=281	N=329	N= 374	N=351	N=268	N=326	N=374
0-5%	55 (15.7%)	115 (40.9%)	70 (21.3%)	89 (23.8%)	58 (16.5%)	116 (43.3%)	74 (22.7%)	105 (28.1%
6-24%	111 (31.7%)	114 (40.6%)	89 (27.1%)	124 (33.2%)	111 (31.6%)	104 (38.8%)	81 (24.8%)	94 (25.1%)
25-49%	133 (38.0%)	48 (17.1%)	104 (31.6%)	121 (32.4%)	136 (38.7%)	44 (16.1%)	98 (30.1%)	145 (38.8%)
50-74%	46 (13.1%)	4 (1.4%)	57 (17.3%)	40 (10.7%)	44 (12.5%)	4 (1.5%)	66 (20.2%)	30 (8.0%)
75-100%	5 (1.4%)	0 (0.0%)	9 (2.7%)	0 (0,0%)	2 (0.6%)	0 (0.0%)	7 (1.1%)	0 (0.0%)
Kneeling/squatting	N=338	N=278		N=374	N=351	N=259		N=374
0-5%	101 (29.9%)	117 (42.1%)		154 (41.2%	119 (31.8%)	119 (31.8%)		158 (42.2%
6-24%	129 (38.2%)	105 (37.8%)		128 (33.7%)	147 (39.3%)	96 (25.7%)		138 (36.9%)
25-49%	80 (23.7%)	39 (14.0%)		67 (17.9%)	56 (15.0%)	33 (8.8%)		50 (13.4%)
50-74%	19 (5.6%)	15 (5.4)		19 (5.1%)	26 (7.0%)	9 (2.4%)		23 (6.1%)
75-100%	9 (2.7%)	2 (0.7)		8 (2.1%)	3 (0.8%)	2 (0.5%)		5 (1.3%)

Table S4.2. Agreement on exposure categories between the Finnish, Norwegian, Swedish and the Nordic JEM. Number of occupational codes in the national JEMs having the same exposure category in both the national JEM and NordicJEM.

		Men			Women	
	Finnish JEM	Norwegian JEM	Swedish JEM	Finnish JEM	Norwegian JEM	Swedish JEM
Exposure/categories	N (%)	N (%)	N (%)	N (%)	N (%)	N (%)
Heavy lifting						
0-5%	87 (24.8)	99 (34.9)	103 (31.2)	112 (32.3)	109 (41.3)	132 (40.6)
6-24%	96 (27.4)	76 (26.8)	65 (18.7)	105 (30.3)	73 (27.7)	71 (21.8)
25-49%	72 (20.5)	41 (14.4)	58 (17.6)	40 (11.5)	27 (10.2)	28 (8.6)
50-74%	8 (1.7)	5 (1.8%)	5 (1.5)	2 (0.6)	1 (0.4)	1 (0.3)
75-100%	2 (0.6)	0 (0.0%)	2 (0.6)	0 (0.0)	0 (0.0)	0 (0.0)
Accuracy	265 (75.5)	221 (77.8)	233 (70.6%)	259 (74.6)	210 (79.5%)	232 (71.4)
Kappa	0.64	0.67	0.58	0.61	0.67	0.55
Fast breathing						
0-5%		67 (23.9)	75 (23.9)		77 (28.5)	95 (30.4)
6-24%		91 (32.5)	80 (25.5)		90 (33.3)	79 (25.2)
25-49%		42 (15.0)	64 (20.4)		40 (14.8)	40 (12.8)
50-74%		14 (5.0)	22 (7.0)		11 (4.1)	7 (2.2)
75-100%		0 (0.0)	0 (0.0)		0 (0.0)	3 (1.2)
Accuracy		214 (76.4)	241 (76.6)		218 (80.7)	224 (71.8)
Kappa		0.66	0.68		0.72	0.58
Forward bent posture						
0-5%	47 (13.4)	70 (24.9)	59 (17.6)	55 (15.7)	80 (29.9)	67 (20.6)
6-24%	68 (19.4)	60 (21.4)	69 (21.0)	58 (16.5)	43 (16.0)	56 (17.2)
25-49%	86 (24.6)	28 (10.0)	74 (22.5)	102 (29.1)	37 (13.8)	79 (24.2)
50-74%	21 (6.0)	4 (1.4)	31 (9.4)	19 (5.4)	2 (0.7)	22 (6.7)
75-100%	0 (0.0)	0 (0.0)	0 (0.0)	0 (0.0)	0 (0.0)	0 (0.0)
Accuracy	222 (63.4)	162 (57.7)	233 (71.5)	234 (66.7%)	162 (60.4)	224 (68.7)
Kappa	0.49	0.39	0.60	0.53	0.43	0.58
Kneeling/squatting						
0-5%	99 (29.3)	98 (35.3)		108 (30.8)	98 (37.8)	
6-24%	88 (26.0)	67 (24.1)		109 (31.1)	61 (23.6)	
25-49%	54 (16.0)	30 (10.8)		40 (11.4)	19 (7.3)	
50-74%	11 (3.3)	9 (3.2)		17 (4.8)	7 (2.7)	
75-100%	5 (1.5)	2 (0.7)		1 (0.3)	2 (0.8)	
Accuracy	255 (75.4)	206 (74.1)		275 (78.3)	187 (72.2%)	
Kappa	0.66	0.61		0.68	0.55	

Supplementary Material 5.

Table S5. Number of occupations in EuroJEM by proportion of exposed workers among men and women.

	Proportion of exposed (%)									
	Men					Women				
Exposures	0-5	6-24	25-49	50-74	75-100	0-5	6-24	25-49	50-74	75-100
Heavy lifting	153	131	77	11	2	177	135	55	7	0
Fast breathing	129	113	107	25	0	141	108/107[1]	110/111	16	0
Forward bent posture	96	113/112[1]	133/134	30	1	102	103/102	139/140	30	1
Hands above shoulder level	139	158	62	12	3	135	163	64	10	2
Kneeling/squatting	158/157	140	52/53	17	8	160/159	142/143	47	19	7

[1] the first number corresponds to the Nordic countries; the second number corresponds to France. In both genders, the differences in categories of forward bent posture and kneeling/squatting were found for "Nursing and midwifery professionals" (code 2230) and "Cooks" (code 5122), respectively. In addition, differences in categories of fast breathing were found for female cooks.
